# Recovery of Waste Material from Biobags: 3D Printing Process and Thermo-Mechanical Characteristics in Comparison to Virgin and Composite Matrices

**DOI:** 10.3390/polym14101943

**Published:** 2022-05-10

**Authors:** Antonella Patti, Stefano Acierno, Gianluca Cicala, Mauro Zarrelli, Domenico Acierno

**Affiliations:** 1 Department of Civil Engineering and Architecture (DICAr), University of Catania, Viale Andrea Doria 6, 95125 Catania, Italy; antonella.patti@unict.it (A.P.); gianluca.cicala@unict.it (G.C.); 2 Department of Engineering, University of Sannio, Piazza Roma 21, 82100 Benevento, Italy; stefano.acierno@unisannio.it; 3 Institute of Polymers, Composites and Biomaterials, Research National Council, P. le Enrico Fermi 1, 80055 Naples, Italy; mauro.zarrelli@cnr.it; 4 Regional Center of Competence New Technologies for Productive Activities Scarl, Via Nuova Agnano 11, 80125 Naples, Italy

**Keywords:** polylactide acid, recycling, film packaging, printing process, wood biocomposites, thermo-mechanical performances

## Abstract

The purpose of this study is to limit the environmental impact of packaging applications by promoting the recycling of waste products and the use of sustainable materials in additive manufacturing technology. To this end, a commercial polylactide acid (PLA)-based filament derived from waste production of bio-bags is herein considered. For reference, a filament using virgin PLA and one using a wood-based biocomposite were characterized as well. Preliminary testing involved infrared spectroscopy, differential scanning calorimetry (DSC), and thermogravimetric analysis (TGA). The effect of printing parameters (namely bed temperature, layer thickness, top surface layers, retraction speed, and distance) on the final aesthetics of 3D printed parts was verified. The results allow us to attest that the thermal properties of recycled polymer are comparable to those of virgin PLA and biocomposite. In the case of recycled polymer, after the extrusion temperature, bed temperature, and printing speed are estabilished the lowest allowable layer thickness and an appropriate choice of retraction movements are required in order to realize 3D-printed objects without morphological defects visible to the naked eyes. In the case of wood biocomposite, the printing process was complicated by frequent obstructions, and in none of the operating conditions was it possible to obtain an aesthetically satisfying piece of the chosen geometry (Lego-type bricks) Finally, mechanical testing on the 3D printed parts of each system showed that the recycled PLA behaves similarly to virgin and wood/PLA filaments.

## 1. Introduction

Conventional packaging is designed as a means of containment, protection, and preservation, with the practical function of holding goods together and protecting them throughout the supply chain until they reach the end user [1].

Thanks to their low cost and multiple benefits, such as lightness, flexibility, and sealability, the majority of currently used packaging plastics are made from fossil resources. As a result, in addition to the inconvenience of not being sustainable, because the planet’s resources are limited [2] such products are not biodegradable and cause environmental problems due to the accumulation of waste material in their surroundings. In 2015, packaging represented 39.9% of the total plastics demand in Europe, accounting for 49 million tonnes of produced plastics, followed by building construction (19.7%), the automotive sector (8.9%), and electronics (5.8%). Furthermore, this field has long been the primary producer of plastic waste, accounting for 59% of total plastic waste generation. It has been reported that the production of plastics and their incineration as waste produces approximately 400 million tonnes of CO_2_ per year globally, and very large quantities of plastic waste leak into the environment both on land and in the oceans. In this context, the European Commission proposes greater circular economy and resource efficiency to support the needs of reuse, repair, and recycling while helping to reduce greenhouse gas emissions and reliance on imported fossil fuels. Voluntary commitment can support ambitious targets, prevent plastics from leaching into the environment, and increase the reuse and recycling of plastic packaging waste (“Plastics 2030”) [3].

The potential and challenges of using biodegradable polymers derived from renewable resources in large-scale food packaging applications have been discussed in previous reviews [4]. Polylactide acid (PLA) is a biodegradable thermoplastic polyester made up of lactic acid monomers. Its synthesis from renewable feedstock using microorganisms has received a great deal of attention [5]. Controlling the monomer composition of the two optical isomeric forms, L and D, allows for the tailoring of properties such as crystallinity, melting temperature, and glass transition temperature. In this manner, different resin grades have been obtained, resulting in a diverse range of products [4]. Polylactide acid (PLA) is often used in food packaging [6], textiles [7], modern medicine [8], and additive manufacturing (AM) [9]. This recent technology, well-known as 3D printing, is emerging as a crucial industrial technology for Rapid Prototyping, allowing a numerical model to be converted into material deposition and 3D printed parts. Prototypes in architecture, DIY (do it yourself) products, and models for automotive or design products have been conceived with PLA polymer considering its excellent biocompatibility and environmental sustainability, absence of unpleasant odours during handling, and production of final products with fair precision tolerance.

In order to improve the physical and chemical properties of PLA polymer, the use of natural polymers (polysaccharides and proteins) extracted from plants or obtained from biomasses or agricultural/industrial wastes are an emerging approach. The benefits of using these natural materials as fillers for 3D printing are due to their availability in nature; biomass/lignin residues can be obtained from the food, pulp and paper, forestry, and agriculture industries. Their introduction in biocomposites contributes to reducing the cost, and limiting the environmental impact of 3D printed specimens by increasing the biodegradability characteristics of final products and promoting the circular economy [10,11].

However, these alternative polymers and composites have this far proven difficult to use in comparison to the standard filament polymers, and few studies have been performed to test their mechanical properties [12]. As reported in a recent review by Khan et al. [13], very limited literature is available related to the development and characterization of 3D printed wood polymer composites. In that study, the tensile properties and impact Charpy strength of two commercial wood–plastic composite materials were analysed in comparison to those of specimens made from pure polylactic acid. The parts were obtained via 3D printing by changing the infill density from 23% to 100%. According to the results, the strength of wood-filled filaments was approximately 40% lower than the strength of the pure PLA specimens. For this reason, such composites have been proposed for low-stress applications such as visualization of prototypes and models, or as decorative items [14]. The effect of wood content up to 50% in wt. added to PLA polymer on the properties of 3D printed parts was investigated in the work of Kariz et al. [15]. Their results showed a decrement in storage modulus when increasing the wood content in 3D printed parts. The glass transition of basic polymer was slightly affected by the wood content. By increasing the fiber percentage in the matrix, other disadvantages such as roughness of surface and worsening of processability arose, and the clustering of wood particles caused clogging in the printer nozzle, hindering the material flow.

The most common 3D printing technique is fused deposition modelling (FDM), in which filament is pushed through a wheel in the extrusion head, melted, and printed into a three-dimensional structure following a geometric pattern using layer-by-layer deposition [16]. Different studies have been performed in the literature comparing the physical and mechanical characteristics of commercial 3D printing PLA grades. Bermudez et al. [17] presented a comparison in terms of mechanical properties, rheological characteristics, chemical qualities, and crystallinity between two grades of commercially available PLA, namely, a widely used filament (PLA Grade 4043D) and a newer grade (3D870). The tensile strength of 3D printed parts made from different commercial filaments (acrylonitrile butadiene styrene, polycarbonate, nylon, T-SemiFlex, Ninjaflex) using an open-source 3D printer was analyzed in [18]. A comparison among the thermal and rheological features of commercially available polylactic acid (PLA) filaments for 3D printing applications presenting different colors (gray, transparent, orange, and natural) was shown in [19], with the blue filament identified as the best in terms of print quality, high-temperature degradation, and thermal stability.

Although the working principle of 3D printing appear to be simple, the FDM method is a complex process in which several parameters (material filament, bed temperature, nozzle temperature and nozzle diameter, filament feed rate, layer thickness, air gap, raster angle and width, fill style, etc.) affect the product quality and material properties [20]. By increasing layer thickness and decreasing the printing temperature, a better surface quality in PLA-based specimens can be achieved [21]. Internal defects and binding strength between layers can be improved by processing the PEEK polymer at higher temperatures and by decreasing the layer thickness and printing speed [22]. As the printing temperature increases, the mechanical properties of 3D printed parts made from PLA and polyethylene terephthalate glycol (PETG) increase; however, an opposite trend occurs when changing the printing speed. In fact, as the printing speed increases, the mechanical properties of the PLA-based specimens increase, while those of PETG-based specimens decrease [23]. It appears clear that each printing parameter is linked to the others, making it challenging to understand the required 3D printing setup for each specific adopted material [20].

In the light of the huge amount of plastic waste resulting from packaging applications and of the wide spread of AM technology, this study seeks to promote the sustainability of processes and products deriving from film packaging by recovering waste materials and reusing them in the 3D printing industry. Recycling polymers used in FDM processes can extend the life cycle of plastic materials, and promote an eco-friendly approach to 3D printing technology [24]. However, during the recycling process plastic materials can undergo thermal degradation. Impurities due to these materials’ use can promote chain-scission reactions by lowering the molecular weight of the polymer and the physical and mechanical features of the corresponding products [24]. In this regard, a commercially available filament from waste recycling of biobags has been considered and characterized through thermal analysis (thermogravimetric analysis, TGA and differential scanning calorimetry DSC) and infrared spectroscopy (ATR). Several printing attempts have been made using variations in the printing process parameters (i.e., bed temperature, layer thickness, top surface layers, retraction speed and distance) to achieve a satisfactory quality of the final 3D printing products without evident macroscopic defects and imperfections appreciable to the eye [25,26,27,28,29] (i.e., poor surface finish, stringing, oozing, delamination, wrapping, misalignment of the print platform and nozzle, clogging of the nozzle, depletion of printing material or disrupted material flow, and lack or loss of adhesion to the print platform). The thermo-mechanical properties of 3D printed parts were developed under optimal printing conditions and measured through dynamic mechanical analysis. The same preliminary characterization carried out for recycled filament along with the optimization of printing parameters was carried out for two other different filaments, namely, a virgin PLA and a wood-based biocomposite. The final performance of the 3D printed parts made from the recycled filament was compared to that of parts made with the virgin polymer and biocomposite.

## 2. Materials and Methods

### 2.1. Materials

Three commercially available poly(lactide) acid (PLA)-based filaments were used in this study. All filaments were supplied by Eumakers (Barletta, Italy).

The first filament source was waste from the production of biobags, utilized in waste collection and segregation, referred to as “recycled PLA”. The biobags are made of biaxially oriented films available in pellet form (cod. Ingeo Biopolymer 4043D, Naturework, Minnetonka, MN, USA) produced by Sfregola Materie Plastiche (Barletta, Italy); the waste from their production was reworked to create a filament suitable for 3D printing.

The second filament was made from virgin PLA-matrix, here identified as “neat PLA”. The third filament was a compound made from PLA and Fir wood fibres, here called “PLA+Wood”.

### 2.2. Sample Preparation

**Figure 1:**
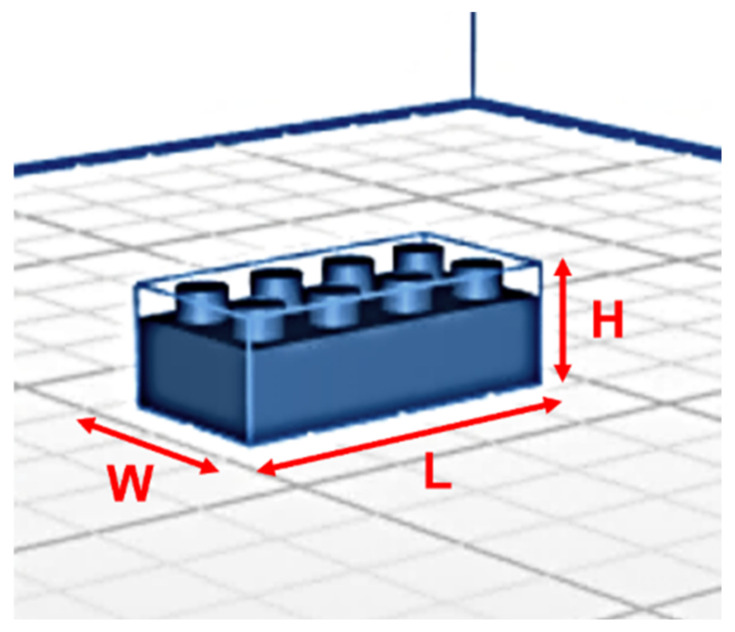
CAD model of the reproduced Lego-type brick (W—width, L—length, H—height).

Filaments were processed through fused deposition modeling (FDM) technology in a 3D printer (cod. M200), produced by Zortax (Olsztyn, Poland). In order to establish an effective set of printing parameters, the material was extruded under various conditions; the appearance of the final products was evaluated by looking for macroscopic defects or imperfections visible to the naked eye and by measuring their size with a caliber. Using a specific CAD model and by setting various technological variables such as bed temperature (60, 65, 70 °C), layer thickness (0.19, 0.14, 0.09 mm), number of constituent layers the top surface (4, 7, 10), retraction speed (36, 27, 26, 24, 20 mm/s), and retraction distance (1.2, 2.2, 2.7 mm), a Lego-type brick with a size of 32 (L) × 16 (W) × 10.8 (H) mm^3^ (Figure 1) was reproduced. A nozzle 0.4 mm in diameter was adopted; Z-Suite was used as the printer management software. Before printing and testing, the material was dried in an oven at 70 °C for 5 h. The printing speed was maintained at a fixed default value (100 mm/s). A linear design pattern and infill density of 100% were always considered.

### 2.3. Characterization Techniques

#### 2.3.1. Attenuated Infrared Spectroscopy (ATR)

Infrared spectroscopy was performed in attenuated total reflectance (ATR) mode using a spectrometer (model Spectrum 65 FT IR) endowed with a diamond crystal, manufactured by Perkin Elmer (Waltham, MA, USA); a wavenumber range of 650–4000 cm^−1^, a resolution of 4 cm^−1^, and a scan number of 16 were chosen. ATR analyses were repeated three times for each material, and a representative curve halfway between the other two measurements was chosen. Each curve was normalized to the peak at 1455 cm^−1^, which is typically considered an internal standard for the PLA polymer as it is associated with the asymmetric bending of the methyl group (CH_3_) [30].

#### 2.3.2. Different Scanning Calorimetry (DSC)

Differential calorimetric tests were performed with a Q2000 DSC (TA Instruments, New Castle, DE, USA) under nitrogen atmosphere. A small sample of polymer (about 8–10 mg in mass) was subject to thermal cycle consisting of: (i) heating (first run) at 10 °C/min from 30 °C to 200 °C in order to assess the material’s properties during the processing conditions; (ii) cooling (second run) at a constant rate of 10 °C/min up to 30 °C; and (iii) reheating (third run) at the same rate up to 200 °C at 10 °C/min in order to evaluate the inherent properties of the material. During the experiment, the melting (T_m_) and crystallization (T_c_) temperatures and corresponding enthalpies of fusion (ΔH_f_) and crystallization (ΔH_c_) were determined. The glass transition temperature (T_g_) was measured at the half-height point of the corresponding heating step [31].

#### 2.3.3. Thermogravimetric Analysis (TGA)

Thermogravimetric measurements were performed using a Q500 TGA (TA Instruments, New Castle, DE, USA). A small piece of material (about 10 mg) was heated at a rate of 10 °C/min from room temperature to 800 °C in both air and nitrogen atmospheres. The initial decomposition temperature (T_dec_) was chosen to correspond to 5% mass loss [32], with residue at 800°C (R_800_) the remaining carbonaceous material at the highest testing temperature that could not be further decomposed into volatile components. The temperature corresponding to the maximum decomposition rate (T_max_) was taken as the peak of the first derivative weight curve (%/°C) [32].

#### 2.3.4. Dynamic Mechanical Analysis (DMA)

A Triton Technology Ltd. (Leicestershire, UK) instrument (mod. Tritec 2000) was used to investigate the dynamic–mechanical properties (DMA) of PLA filaments. Rectangular specimens (nominal size of 2 × 5 × 25 mm^3^, support distance of 12 mm) were prepared by 3D printer in optimized conditions and tested in single cantilever mode at a frequency of 1 Hz and temperatures ranging from room temperature to 70 °C. or each material and each condition, the test was repeated three times, using different samples, in order to verify date reproducibility.

## 3. Results

### 3.1. Qualitatively Analysis of Basic Constituents (ATR)

**Figure 2:**
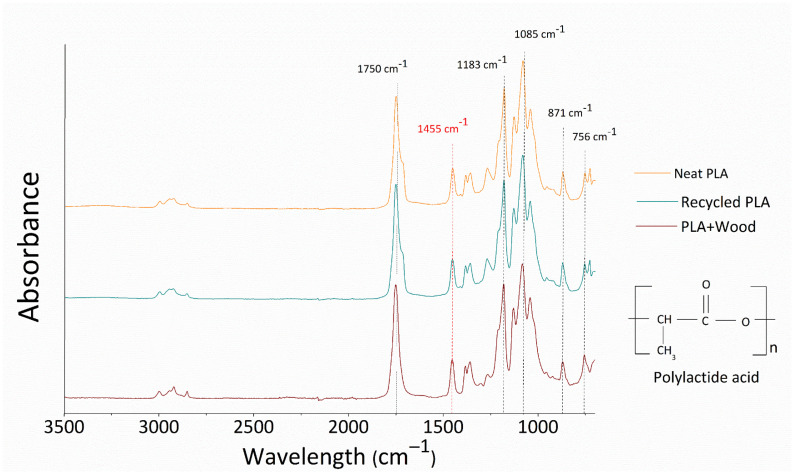
ATR spectra of investigated filaments.

A graphical comparison of the ATR spectra of the investigated specimens in terms of absorbance versus wavelength (cm^−1^) is shown in Figure 2.

In each spectrum, the common peaks of PLA can be recognized in the polymer fingerprint. At 1750 cm^−1^, the peak attributed to carbonyl (C=O) stretching was identified. Then, at 1183 cm^−1^ and 1085 cm^−1^ two peaks attributed to the asymmetric vibration of the ester group (C–O–C) were distinguished [33,34].

In previous studies, the same peaks have usually been used as a reference for identifying the thermal oxidation/degradation of PLA based-matrices [30]. Overall, there were no significant differences between the spectra of the three investigated materials. This indicates that the degradation phenomena in the recycled filament were negligible or absent when compared to those in the common virgin polymer. In Table 1, absorbance values at the above-mentioned characteristic peaks are shown for each material.

According to the work of Pop et al. [35], the two bands at 871 and 756 cm^−1^ can be attributed to PLA’s amorphous and crystalline phases, respectively. The PLA’s crystallinity index can be calculated through the ratio of the areas underlying the characteristic peaks at 871 and 756 cm^−1^ (Area_(756)_/Area_(871),_ shown in Table 1).

Taking into account this consideration, the neat and recycled PLA filaments showed adsorption peaks at 756 cm^−1^ and 871 cm^−1^ that were nearly comparable in size. On the contrary, the PLA+Wood spectrum shows a greater absorbance at 756 cm^−1^ (and thus a higher area) compared to that at 871 cm^−1^. As a consequence we can speculate that specimens containing wood fibers have a higher index of crystallinity than neat matrices.

As concerns the presence of wood within the PLA+Wood samples, the typical infrared spectrum of wood flour generally shows the predominance of hydroxyl band at 3100 to 3700 cm^−1^ and ether species (C–O–C) at 1000–1150 cm^−1^, then weak bands of carbonyl groups at 1800–1680 cm^−1^, vinyl (C=C) groups at 1650–1500 cm^−1^, and methyl species (CH_3_) at 1450 and 1350 cm^−1^ [36]. In this case, the characteristic peaks attributable to the presence of wood fibers can not be distinguished, implying that the filler is well embedded in the polymer matrix [35] or, alternatively, that the wood content is low and the characteristic peaks are therefore hidden beneath those of the PLA.

### 3.2. Glass Transition and Melting Point (DSC)

**Figure 3:**
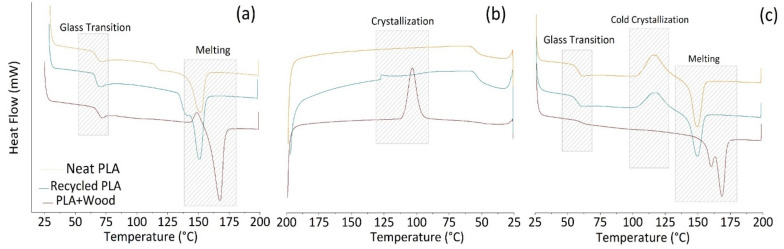
Heat flow vs. temperature recorded during thermal cycles of DSC analyses for the investigated samples: (**a**) first heating; (**b**) cooling; (**c**) second heating.

Thermograms from the calorimetric analyses are reported in Figure 3 in terms of heat flow (mW) vs. temperature (°C) for each investigated material.

During the first scan of non-isothermal DSC (Figure 3a), neat and recycled PLA show a second order transition followed by an endothermic peak. The first phenomenon is typical sign of glass transition (T_g_) around 62–63 °C while the endothermic peak is related to the melting of crystalline regions, with a maximum at 150 °C.

In the second heating scan (Figure 3c), glass transition temperatures are about 5 °C lower in both cases compared to those recorded in the first heating, while the melting point remain at the same temperature (~150 °C).

The crystallization phenomenon was not visible in either the form of cold crystallization during the first heating scan or during the cooling phase. In this respect, it should be mentioned that the samples subjected to DSC analysis were previously dried for 5 h at a temperature of 70 °C. As a consequence, it is possible that the cold crystallization has occurred during this phase and therefore was no longer visible in the first heating scan. A similar effect has been reported in the case of thermal annealing performed for 1 h in an oven at 105 °C in a study by Perez-Fonseca et al. [37]. In their work, the cold crystallization peaks were not observed for thermal treated samples with respect to untreated ones, indicating that the crystalline region in the system may be undetectable due to the crystallinity being modified during thermal annealing.

Then, during the second run, it might be the case that the “standard” cooling rate (10 °C/min) was too fast compared to the low crystallization kinetics of PLA macromolecules.

As reported by Wang et al., the L-isomeric form of PLA chains is typically the primary component of commercially available PLA pellets. The incorporation of D-lactic acid into PLA molecules reduces crystallinity and spherulite growth rates.

PLA containing 4–8% of D-isomer contents would be more suitable for blow molding in order to limit the rapid crystallization of the polymer and avoid the stretching of the preform and optical clarity of the products [38].

As the chosen filaments were commercially available, we are not able to exclude the combination of L- and D-isomers in order to develop specific features in the PLA-based materials that could limit the crystallization rate.

As concerns the DSC data for PLA+Wood samples, during the first heating the glass transition takes place at around 69 °C and the cold crystallization peak (149 °C) is very close to the melting peak (167 °C). During the second heating, the glass transition temperature takes places at a lower temperature (60 °C) and the melting shows two separate peaks (at 159 and 168 °C). In general, PLA has a variety of crystalline forms (α, β, γ, δ); the common crystallite α-form is obtained from the melt through a slow cooling procedure. The crystalline β-phase is obtained by drawing the PLA at high temperatures. The δ-form is derived by epitaxial crystallization on a single substrate. Finally, the δ-form, referred as imperfect α-form, is observed in samples processed from the melt using a rapid cooling procedure [39]. The presence of two melting temperatures is usually attributed to the melting of the α form and disordered α form (α’), respectively [40].

The higher T_g_ (69 °C) in the PLA+Wood samples compared to PLA-based filaments (~62–63 °C) can be interpreted as a delay in polymer relaxation in the presence of reinforcement due to greater crystallinity [41], as also qualitatively confirmed by the ATR measurements. This consideration, together with the existence of exothermic peaks during the first heating and the cooling phases, can be attributed to nucleating effect of the wood fibers on the PLA macromolecules, promoting crystal formation in PLA-based materials.

Furthermore, in the wood composites, during the first heating, a small exothermic peak can be observed at a relatively high temperature (~149 °C), higher compared to similar studies (102.8–112.9 °C [42]; 109.5–115.9 °C [43]), which is very close to the melting (~167 °C). This behavior could be attributed to a poor thermal stability of early crystallites formed during the drying phase and their re-crystallization near the melting zone. The recrystallization peak indicates that previously-formed crystallites did not achieve the stable orthorhombic α form [44].

Temperatures and enthalpy changes corresponding to the main observed phenomena (i.e., glass transition, melting and crystallization) during the thermal analytical cycle (first heating, cooling, second heating) are summarized in Table 2.

### 3.3. Thermal Degradation

**Figure 4:**
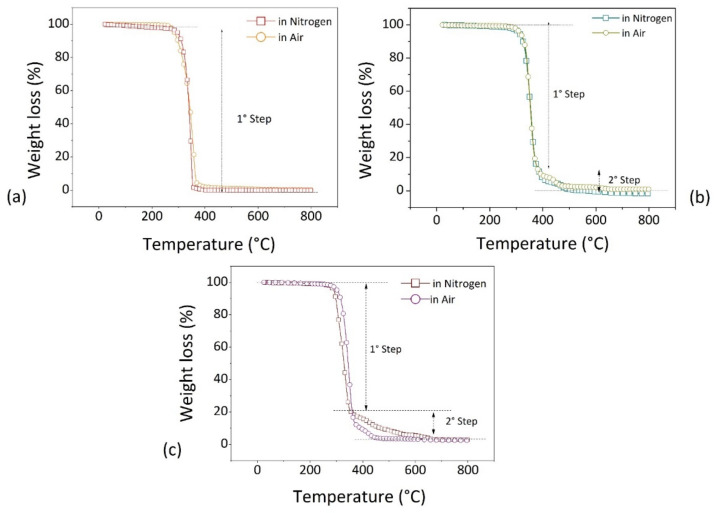
Thermograms of: (**a**) basic PLA; (**b**) Recycled PLA; (**c**) PLA+Wood filaments.

Thermogravimetric analysis was used to investigate the mass loss of PLA-based filaments in both inert (nitrogen) and active (air) atmospheres. The obtained thermograms are reported in Figure 4 for neat PLA filament (Figure 4a), recycled polymer from bio-bags (Figure 4b), and wood composite (Figure 4c).

Depending on the testing atmosphere, different thermal degradation phenomena can occur. In nitrogen atmosphere, pyrolysis develops through a series of reactions such as depolymerization, thermal cracking, and ring-opening. During pyrolysis, long macromolecules are converted into other components with low molecular weights. In an oxygen environment, gasification and combustion take place and pyrolysis is reduced to substeps that produce primarily gas and char [45]. Most polymers burn in aerobic conditions and transforms in water (H_2_O), carbon dioxide (CO_2_), and volatile components [46].

For the neat PLA (Figure 4a), one single step of thermal degradation is visible leaving an amount of residue char at 800 °C equal to zero. This step can be attributed to the mechanisms of random chain scission or specific chain-end scission, as the repeated aliphatic ester structure of PLA polymer is hydrolyzed and broken relatively easy [47]. When comparing data from the two different environments, the thermogram moves to a slightly lower temperature (the onset decomposition temperature is 296 °C in the nitrogen atmosphere and 285 °C in the air atmosphere).

For the recycled filament (Figure 4b), the degradation curves recorded under air and nitrogen atmospheres are very similar. In both cases the material shows a two-step thermal degradation behavior, with a final residue at 800 °C equal to zero. This result is different from that reported in the work of Olejnik and Masek [48] for neat pellets (IngeoTM Biopolymer 4043D). In their case, the thermal degradation attested to a unique stage with an onset at temperature around 340 °C. This led them hypothesize the presence of another species added during the preparation of recycled PLA after the recovery of waste film packaging beyond basic pellets, declared as main constituents of bio-bags.

In the case of PLA+Wood filament (Figure 4c), the thermal degradation happens in two stages. The first step takes place in the temperature range between 290 and 370 °C, with the material that decomposes faster in nitrogen atmosphere. The second step takes place in the range between 370 and 650 °C, with an opposite behavior with respect of the first step and the material showing a faster disintegration rate in air.

In the literature, thermogravimetric analysis of wood and wood compounds reveals three stages of decomposition, consisting of dehydration (water evaporation), active pyrolysis (main devolatilization), and passive pyrolysis [49]. In active pyrolysis, hemicelluloses and cellulose decompose in temperature ranges from 473 to 653 K (~200–380 °C) and 523 to 653 K (~250–380 °C), respectively. On the contrary, lignin decomposes during both active and passive pyrolysis in temperature range from 453 to 1173 K (180~900 °C), without characteristic zones [50].

Taking this into account, we conclude that the final residue at 800 °C (~2.5–2.8%) measured at the end of the tests, under both air and nitrogen atmospheres, is due to remaining parts of the undecomposed lignin present in the wood.

The good resistance of wood compounds under heating can be attributed to the formation of protective barriers of char that inhibit the thermo-oxidation process [36,51]. This behavior is coherent with the formation of tortuous pathways of dispersed fibers in the polymer, which slows down the mass diffusion of degradation products from the bulk polymer to the gas phase, reduces overall thermal conductivity, and protects the material from thermal decomposition [32,36]. As concerns the recycled filament, the presence of another component introduced into the matrix during the recovery of waste products for the purpose of increasing resistance under mechanical and/or heat stress can be reasonably supposed in light of its being a commercial formulation. The presence of this species has been hypothesized after comparing the data on the weight loss of the recycled filament against temperature with the data reported in the literature [48]. We recorded a two-step of thermal degradation rather than the single step in the work of Olejnik et al. for the same basic material used to make biobags (Ingeo Biopolymer 4043D) [48]. This can be interpreted as an evidence of the presence of another component in addition to the basic ones in the recycled formulation. This can be due to two reasons: (i) the introduced quantities of the second phase were insufficiently detected by the IR and DSC analyses; (ii) the second species possessed the same functional groups and similar thermal characteristics as the first component, namely, PLA polymer.

Table 3 summarizes the TGA results for the investigated materials.

### 3.4. Printing Parameters for Recycled PLA

**Figure 5:**
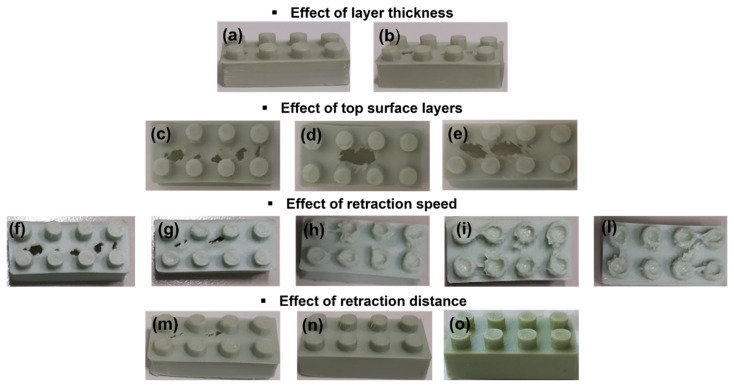
Effect of printing parameters on the final aesthetics of 3D piece made from recycled PLA corresponding to the conditions reported in Table 4 ((**a**)—layer thickness of 0.19 mm, (**b**)—layer thickness of 0.09 mm, (**c**)—top surface layers equal to 4, (**d**)—top surface layers equal to 7, (**e**)—top surface layers equal to 10, (**f**)—retraction speed of 36 mm/s, (**g**)—retraction speed of 27 mm/s, (**h**)—retraction speed of 26 mm/s, (**i**)—retraction speed of 24 mm/s, (**l**)—retraction speed of 20 mm/s, (**m**)—retraction distance of 1.2 mm, (**n**)—retraction distance of 2.2 mm, (**o**)—retraction distance of 2.7 mm).

All printing attempts made to determine the best printing quality in the case of recycled PLA are summarized in Table 4. The effects of the various 3D printing parameters on the final aesthetics of the 3D printed Lego-type bricks are shown in Figure 5. Each printing condition indicated in Table 4 is associated with an image of the final sample in Figure 5.

#### 3.4.1. Platform (or Bed) Temperature

The bed temperature was the first parameter studied for the 3D printing process. This should be set higher than the feedstock material’s glass transition temperature in order to ensure that the first layer of extruded filament adheres completely to the building base (bed). However, this temperature should not be too high, in order to avoid printing object distortion during removal from the bed [52]. No systematic studies have revealed strategies for identifying an optimal printing bed temperature range for the various materials or the corresponding effects on sample adhesion in FDM technology. In the case of PLA and acrylonitrile butadiene styrene (ABS), it was discovered that heating the printing bed slightly above the T_g_ of the filament material result in optimal adhesion of the printed sample to the printer platform [52]. Increasing the temperature above the T_g_ reduces the surface tension between the platform and the extruded material, resulting in a larger contact area and better adhesion of the polymer to the printer support [52].

Attempts to identify the best platform temperature demonstrated first-layer adhesion to the base at temperatures higher than 10 °C with respect to the glass transition point of recycled polymer.

#### 3.4.2. Layer Thickness and Top Surface Layers

The second analyzed parameter was the layer thickness, i.e., the height of the detached material layers from one another during the layer-by-layer deposition process. This parameter is considered the most important in affecting the surface roughness, and therefore the precision and printing quality [53]. Increasing the layer thickness reduced the number of layers required to print the part. As the number of layers decreased, the motion of the nozzle head was reduced, leading to a reduction in the build time. Thinner layer thicknesses and higher numbers of slices constituting the perimeter of 3D objects led to higher time to print the part [54]. On the one hand, decreasing the layer thickness led to a higher required printing time; on the other hand, a practical demonstration allowed us to verify a gain in printing precision, with more accurate edges and less perceptible separation between layers. In fact, by reducing the layer thickness from 0.19 mm (Figure 5a) to 0.09 mm (Figure 5b) (the lowest limit of the 3D printer used in this study), the distinction between layers became less visible and the edges were more defined; however, the surface of the specimen appeared increasingly perforated.

Top surface layers are the number of layers used to cover the upper surface of the empty form. A practical demonstration allowed us to verify that when increasing this value from 4 (Figure 5c) to 7 (Figure 5d) up to 10 (Figure 5e) the sample surface presented holes of increasing size.

#### 3.4.3. Retraction Speed and Distance

Retraction movements are performed counter-clockwise in order to avoid oozing during the movement of the print head, during which the melt should not escape. In detail, this is a backwards movement that brings the material back with the goal of lowering the pressure at the tip of the nozzle and preventing oozing during non-printing movements. Similarly, before beginning to print an opposing movement is performed in order to renew the pressure at the extrusion system’s tip. Retraction is defined through two parameters, namely, length (retraction distance) and speed (retraction speed) [55].

On the other hand, the printing speed indicates how fast the printer moves during material deposition [55].

Retraction movements can determine quantity and deposition of the material, affecting sample size, perimeter, and printing quality. In detail, a lower retraction speed means a higher material flow during non-printing movements, a larger perimeter of minute particulars, and more evident oozing and stringing phenomena. On the other hand, by increasing the retraction distance it is possible to balance the quantity of exuded material by reducing morphological defects [55] and avoiding holes on the upper surface.

In this case, the printing speed was fixed at a default value of 100 mm/s, whereas the retraction speed was changed from 36 mm/s (Figure 5f) to 20 mm/s (the lowest allowable limit) (Figure 5l).

As highlighted in Figure 5, when decreasing the retraction speed, the sample surface no longer appeared to be pierced, however, too much material accumulated on it.

At a retraction speed equal to or lower than 26 mm/s (Figure 5h,i,l), the surface was uniformly covered while precision in fine details was lost. More material was not held during no-printing movements, and much more material accumulated on the piece’s small circular points (upper cylinders) or in the intermediate space between them.

The effect of retraction distance was the last aspect considered in setting the printing conditions. The retraction distance is the length of filament that is pulled back by the extruder every time retraction occurs. A value of 1.2 mm (Figure 5m) was fixed as the starting point and then increased to 2.2 mm (Figure 5n), then to 2.7 mm (Figure 5o). the most satisfactory 3D printed parts were obtained with a retraction distance of 2.7 mm.

The main sizes (H, W, L) of the 3D objects were measured as the retraction speed and the retraction distance were changed (Table 5).

In addition to the effect on macroscopically relevant defects, by decreasing the retraction speed from 36 to 20 mm/s the height of the piece (H) on the top surface was increased by 17%; no strong effect of retraction distance was highlighted with respect to the size of the samples.

**Table 5:** Average size and standard deviation value (on di8 points) of Lego-type bricks as a function of changes in retraction speed and distance.

**Retraction Speed** **(mm/s)**	**W** **(mm)**	**L** **(mm)**	**H** **(mm)**
36	15.56 ± 0.21	31.63 ± 0.13	11.87 ± 0.42
27	15.71 ± 0.22	31.53 ± 0.10	11.70 ± 0.40
26	15.61 ± 0.13	31.76 ± 0.09	11.82 ± 0.32
24	15.65 ± 0.13	31.62 ± 0.05	12.53 ± 0.71
20	15.64 ± 0.09	31.67 ± 0.11	12.53 ± 0.47
**Retraction** **Distance (mm)**	**W** **(mm)**	**L** **(mm)**	**H** **(mm)**
1.2	15.71 ± 0.22	31.53 ± 0.10	11.70 ± 0.40
2.2	15.83 ± 0.04	31.71 ± 0.03	11.41 ± 0.03
2.7	15.73 ± 0.09	31.72 ± 0.03	11.73± 0.23

### 3.5. Printing Parameters for Neat PLA

Apart from the extrusion temperature (fixed at 210 °C instead of 190 °C), the printing conditions of Neat PLA were found to be the same as recycled PLA.

Attempts to print the Neat PLA at 190 °C revealed significant warping defects.

This deformation is a common problem with FDM technologies and is attributed to the thermal gradients involved in the printing process, which can cause layer separation, deformation, and curling of corners [56]. Following deposition on the platform, the extruded polymer cools and contracts while the hot nozzle moves around the build [56].

It is possible that a nozzle temperature of 190 °C causes faster cooling than a temperature of 210 °C, particularly for the first layers deposited directly on the platform at 70 °C. Furthermore, the material’s viscosity is predictably higher at 190 °C than at 210 °C, leading to lower interpenetration of one stratum on the other. This in turn causes tension within the object, and when the tension becomes too great the lower layers begin to drag or lift, resulting in a wrap.

### 3.6. Printing Parameters for PLA+Wood

Several attempts were required to print the composite filament due to frequent blockages of the duct, with the result that a satisfactory final piece was never obtained.

The retraction speed was reduced as much as possible, up to 20 mm/s, and layer thickness was increased to 0.19 mm in order to facilitate material movement and avoid obstruction. However, stringing and oozing defects were always present in the final piece, and were generated corresponding to retraction distances of less than 1 mm. A value of this parameter greater than 1 blocked the nozzle and prevented the material from being printed.

Computational fluid dynamics and discrete element method, as developed in the work of Zhang et al. [57], have confirmed a strong correlation between fibre length and filler volume fraction. In the case of a nozzle diameter of 0.45 mm, a short fibre length of 0.24 mm was found to effectively avoid clogging if the wood content was not higher than 26.68%. If longer fibres were present in the polymer (L = 0.35 mm), the material flow in the conduit was hindered by the fiber length regardless of the matrix viscosity. In this condition, the fibre volume fraction should not be greater than 20.01%. Even with a very low fiber volume equal to 13.34%, increases in fiber length up to 0.45 mm resulted in nozzle clogging. Therefore, the combination of two factors, such as fiber length and volume concentration, should be balanced in order to avoid the risk of nozzle clogging.

According to Lewis and Gratson, colloidal gel-based inks require significant applied pressure to induce flow during layer deposition and suffer from clogging when the ratio between diameters of nozzle and particles is reduced to less than 100 [58].

The presence of wood fibers has been found to have a significant impact on printing quality and printer nozzle clogging. This is attributed to the melted composite’s higher viscosity compared to pure polymer as well as to the higher pressure required to push filament through the nozzle [58]. The fibers’ length and content, as well as the process parameters (i.e., printing speed and nozzle diameter), are considered responsible for fiber alignment through the inducing of shear stress through the extrusion nozzle and avoiding blocking of the conduit [57].

### 3.7. Thermo-Mechanical Characteristics of 3D Printed Parts in Optimized Printing Conditions

The final FDM parameters used with the three different filaments to print DMA specimens are summarized in Table 6.

**Figure 6:**
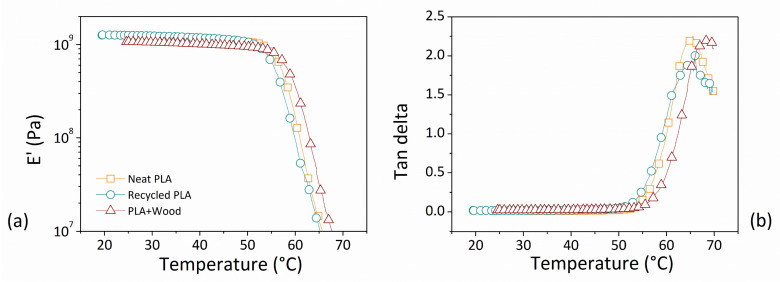
(**a**) Storage modulus (E’) and (**b**) dissipation factor as a function of temperature for investigated materials. (Legend is the same in (**a**) as in (**b**)).

The experimental results of our dynamic–mechanical analysis are reported in Figure 6 in terms of the storage modulus (E’) in Pa (Figure 6a) and dissipation factor (tan delta) vs. the testing temperature (Figure 6b) for the three investigated systems.

The storage modulus (E’) together with the loss modulus (E”) makes up the complex modulus. During one oscillation cycle E’ represents the energy contribution that is preserved, whereas E” represents the lost energy contribution. The dissipation factor expresses the ratio between the lost and stored energies, providing the overall system’s damping ability. The temperature dependence of the storage modulus provides information on changes in the stiffness of the material as a result of thermal variations. The value of tan delta at the maximum point indicates the temperature corresponding to the glass transition (T_g_) [34].

By increasing testing temperature for each material, two different typical zones were identified in the storage modulus trend: the first, during which the modulus remained almost constant, was commonly associated with the glassy zone, and the second, during which a drastic reduction in the studied parameter was verified, was usually typical of the transition from a glassy to a rubbery state.

No strong differences were highlighted in comparing the thermo-mechanical characteristics of the investigated materials. As shown in Table 7, at a temperature of 30 °C the storage modulus of the recycled matrix was almost comparable with that of neat polymer, and a slightly lower value was evaluated corresponding to the composite filament. In this case, the presence of the wood fiber incorporated into the neat matrix seemed not to contribute any effective reinforcement to the mechanical resistance of the final products. This result can be attributed to two distinct aspects, first, the low interfacial bonding between the filler and matrix in light of the hydrophilic nature of the fibers and hydrophobic nature of the polymer, and second, the poor adhesion between layers that occurred in the FDM printed parts when the bottom layer quickly solidified with a molten top layer resting on it [59].

As published in the work of Gupta et al. [60] using DMA scans at 1 Hz with neat PLA specimens made from Ingeo Biopolymer 4043D pellets, the storage modulus curve corresponding to the glassy region remained at around 10^9^ Pa, which is very close to that measured here in recycled filament specimens.

Furthermore, for each filament type the values of the glass transition temperature were fairly close to those determined by DSC. In both analyses, the temperature at the tan delta peak of the composite material filament was always slightly higher than that of the base polymers. Contrary to expectations, the intensity of the tan delta peak for the composite was roughly equal to those of the other two materials. This result indicates the similar ability of the moving polymer chains in both the filled and unfilled systems, and can be attributed to the aforementioned poor filler/matrix interaction. This means that in the composite material, supposing weak filler/matrix interactions, the wood fibers did not interfere with chain movements by preventing damping.

## 4. Conclusions

In this study, three different PLA-based filaments (one made from a basic polymer, another from recycled packaging products, and the last from a combination of wood and PLA) were processed using FDM technology and characterized in terms of their spectroscopic, thermal, and mechanical properties. Infrared analysis demonstrated characteristic adsorption bands typical of polylactide polymer for all the three filaments. Thermogravimetric investigation revealed that the recycled (T_dec_ = 313 °C) and the composite (T_dec_ = 303 °C) materials had greater thermal stability than basic PLA (T_dec_ = 285 °C). The glass transition and melting temperature of recycled PLA were very similar to those of neat PLA (T_g_~60 °C, T_m_~150 °C), and lower than those of the biocomposite (T_g_~69 °C, T_m_~167 °C). The printing process and quality of the final products in terms of macroscopic visible imperfections and sample sizes were found to be strongly affected by layer thickness, retraction speed, and distance. After determining the fixed extruder and platform temperatures and printing speed in the case of neat and recycled PLA and finding an appropriate balance between the layer thickness (the lowest allowable, equal to 0.09 mm) and retraction parameters (a retraction distance of 2.7 mm and retraction speed of 27 mm/s), 3D printed objects (Lego-type bricks) free of morphological defects were obtained. In the case of the wood-based filament, the extrusion process was practicable only when setting the retraction speed to the lowest allowable value (20 mm/s) and increasing the layer thickness (0.19 mm). However, it was not possible to wholly eliminate stringing effects by changing the retraction distance (not greater than 1 mm). Finally, DMA results showed that the average storage modulus of specimens made from recycled PLA (1.20 × 10^9^ Pa) was comparable to that of specimens made from neat polymer (1.13 × 10^9^ Pa), and slightly higher (+15%) than that of filled PLA with wood (1.03 × 10^9^ Pa).

In conclusion, the recovered polymer material from packaging waste can be considered promising for 3D printing applications thanks to its good printability when the proper parameters are used as well as to its thermal and mechanical properties, which are comparable to those of virgin PLA. Furthermore, in order to increase the use of sustainable sources in FDM applications, recycled polymers may represent a preferable solution to wood-based biocomposites in view of the difficult extrudability of the latter system.

## Figures and Tables

**Table 1:** Average absorbance values at specific wavelengths (1750 cm^−1^, 1183 cm^−1^, 1085 cm^−1^, 871 cm^−1^, 756 cm^−1^) and ratio of the areas underlying the characteristic peaks at 871 and 756 cm^−1^.

	A_1750_	A_1183_	A_1085_	A_871_	A_756_	Area_(756)_/Area_(871)_
Neat PLA	3.46 ± 0.44	3.43 ± 0.22	4.19 ± 0.43	0.99 ± 0.08	1.11 ± 0.02	1.15 ± 0.05
Recycled PLA	2.68 ± 0.21	2.75 ± 0.30	3.39 ± 0.40	0.91 ± 0.04	0.89 ± 0.01	0.88 ± 0.01
PLA+Wood	2.54 ± 0.37	2.54 ± 0.35	2.98 ± 0.04	0.97 ± 0.08	1.12 ± 0.06	1.37 ± 0.11

**Table 2:** DSC data for dried filaments during the first and second heating (glass transition, T_g_), cold crystallization (T_cc_) and melting (T_m_) temperatures and corresponding enthalpies (ΔH_cc_, ΔHf), and cooling (crystallization temperature, T_c_) and relative enthalpy (ΔH_cc_).

	1st Heating					Cooling		2nd Heating					
	T_g_ (°C)	T_cc_ (°C)	ΔH_cc_ (J/g)	T_m_	ΔH_f_ (J/g)	T_c_ (°C)	ΔH_c_ (J/g)	T_g_ (°C)	T_cc_ (°C)	ΔH_cc_ (J/g)	T_m1_	T_m2_	ΔH_f_ (J/g)
Neat PLA	63	-	-	150	40.5	-	-	59	117	23.9	149	-	33.4
Recycled PLA	62	-	-	150	41.2	-	-	58	118	20.1	150	-	31.3
PLA+Wood	69	149	3.9	167	45.1	103	37.6	60	-	-	160	168	43.9

**Table 3:** Initial decomposition temperature (T_dec_), temperature corresponding to the maximum rate of decomposition in the first step (T_max1_), temperature corresponding to the maximum rate of decomposition in the second step (T_max2_), remaining mass % of material at highest testing temperature (R800).

	T_dec_	T_max1_	T_max2_	R_800_
In nitrogen atmosphere				
Neat PLA	296 °C	348 °C	-	0%
Recycled PLA	311 °C	359 °C	475 °C	0%
PLA+Wood	290 °C	331°C	433 °C	2.8%
In air atmosphere				
Neat PLA	285 °C	350°C	*-*	0.1%
Recycled PLA	315 °C	355 °C	423 °C	0.8%
PLA+Wood	303 °C	350 °C	450 °C	2.5%

**Table 4:** Attempts to determine the best printing quality in the case of recycled PLA.

Extruder Temperature (°C)	Bed Temperature (°C)	Retraction Speed (mm/s)	Top Surface Layers	Layer Thickness (mm)	Retraction Distance (mm)
Effect of platform temperature					
190	60	36	4	0.19	1.2
190	65	36	4	0.19	1.2
190	70	36	4	0.19	1.2
Effect of layer thickness					
190	70	36	4	0.19	1.2
190	70	36	4	0.14	1.2
190	70	36	6	0.09	1.2
Effect of top surface layer					
190	70	36	4	0.14	1.2
190	70	36	7	0.14	1.2
190	70	36	10	0.14	1.2
Effect of retraction speed					
190	70	36	6	0.09	1.2
190	70	27	6	0.09	1.2
190	70	26	6	0.09	1.2
190	70	24	6	0.09	1.2
190	70	20	6	0.09	1.2
Effect of retraction distance					
190	70	27	6	0.09	1.2
190	70	27	6	0.09	2.2
190	70	27	6	0.09	2.7

**Table 6:** Final processing conditions used to print DMA specimens.

	Neat PLA	Recycled PLA	PLA+Wood
Extruder temperature (°C)	210	190	210
Bed Temperature (°C)	70	70	70
Retraction Speed (mm/s)	27	27	20
Top Surface layers	6	6	6
Layer thickness (mm)	0.09	0.09	0.19
Retraction distance (mm)	2.7	2.7	1

**Table 7:** Storage modulus at 30 °C and glass transition temperature by DMA.

	Storage Modulus at 30 °C (Pa)	Temperature at Tan Delta Peak (°C)
Neat PLA	1.13 × 10^9^ ± 1.10 × 10^8^	65.3 ± 0.3
Recycled PLA	1.20 × 10^9^ ± 5.27 × 10^7^	65.8 ± 1
PLA+Wood	1.03 × 10^9^ ± 3.51 × 10^7^	69.3 ± 0.4

## Data Availability

The data presented in this study are available on request from the corresponding author.
